# Lineage and Virulence of *Streptococcus suis* Serotype 2 Isolates from North America

**DOI:** 10.3201/eid1712.110609

**Published:** 2011-12

**Authors:** Nahuel Fittipaldi, Jiangu Xu, Sonia Lacouture, Prasit Tharavichitkul, Makoto Osaki, Tsutomu Sekizaki, Daisuke Takamatsu, Marcelo Gottschalk

**Affiliations:** Université de Montréal, St-Hyacinthe, Quebec, Canada (N. Fittipaldi, S. Lacouture, M. Gottschalk);; National Institute for Communicable Disease Control and Prevention, Beijing, People’s Republic of China (J. Xu);; Chiang Mai University, Chiang Mai, Thailand (P. Tharavichitkul);; Agriculture, Forestry and Fisheries Research Council, Tokyo, Japan (M. Osaki);; The University of Tokyo, Tokyo (T. Sekizaki);; National Institute of Animal Health, Tsukuba, Japan (D. Takamatsu)

**Keywords:** Streptococcus suis, bacteria, serotype 2, multilocus sequencing typing, MLST, North America, zoonoses, swine, streptococci, lineage, virulence

## Abstract

We performed multilocus sequence typing of 64 North American *Streptococcus suis* serotype 2 porcine isolates. Strains were sequence type (ST) 28 (51%), ST25 (44%), and ST1 (5%). We identified nonrandom associations between STs and expression of the virulence markers suilysin (SLY), muramidase-relased protein (MRP), and extracellular factor (EF). Expression of pili encoded by the *srtF* and *srtG* pilus clusters was also nonrandomly associated with STs. ST1 strains were SLY+ EF+ MRP+ *srtF* pilus+ *srtG* pilus−. ST25 strains were SLY− EF− MRP− *srtF* pilus− *srtG* pilus+, and most ST28 strains were SLY− MRP+ EF− *srtF* pilus+ *srtG* pilus+. ST28 isolates proved essentially nonvirulent in a mouse infection model; ST25 strains showed moderate virulence and ST1 isolates were highly virulent. ST1 is responsible for a high proportion of *S. suis* disease in humans worldwide. Its presence in North America indicates that potential zoonotic *S. suis* outbreaks in this continent cannot be disregarded.

## Abstract 2

Two sequence types predominate and have lower virulence than other types.

*Streptococcus suis* causes meningitis and septicemia in pigs and is a zoonotic agent (*1*). In the Western hemisphere, human *S. suis* disease is infrequent and usually affects workers in the swine industry. However, *S. suis* is the most commonly reported cause of streptococcal meningitis in adults in Vietnam and the second in Thailand (*2**,**3*). Two outbreaks of human *S. suis* disease have occurred in People’s Republic of China, affecting hundreds of persons and causing 39 deaths (*4*). Most cases of animal and human *S. suis* infection have been caused by serotype 2 strains (*5*). The percentage of *S. suis* serotype 2 strains recovered from diseased pigs and the number of cases of human disease is lower in North America than in other parts of the world (*6**,**7*).

Multilocus sequence typing (MLST) has shown that *S. suis* serotype 2 strains can be divided into at least 16 sequence types (STs). Closely related STs are grouped in the so-called ST complexes. Although ST complexes 1, 27, and 87 dominate the *S. suis* population, most invasive isolates belong to the ST1 complex (*8*). For example, most strains isolated from human patients in Japan were ST1 (*9*), whereas those causing the human outbreaks in People’s Republic of China were ST7, included in the ST1 complex (*10**,**11*). However, Takamatsu et al. showed that 80% of the isolates recovered from blood or cerebrospinal fluid of humans in Thailand belonged to STs grouped in the ST27 complex (*12*).

Most of the *S. suis* serotype 2 strains genotyped so far by MLST originated in Europe and Asia (*8**–**12*). Isolates from Canada and the United States have received less attention. In this study, we used MLST to genotype a relatively large collection of US and Canadian *S. suis* serotype 2 strains.

## Materials and Methods

### *S*. *suis* Field Strains

Sixty-four strains of *S. suis* serotype 2 isolated from pigs with clinical disease in different and nonrelated farms in major swine production areas of Canada and the United States were used. For comparison purposes, 19 porcine and 1 human *S. suis* serotype 2 strains isolated in Japan and 12 human *S. suis* serotype 2 strains isolated in Thailand were included (*12**,**13*). All strains are listed in Table A1.

### MLST and Phylogenetic Analysis

*S. suis* genomic DNA was prepared from overnight cultures by using the QIAamp DNA Minikit (QIAGEN, Valencia, CA, USA) following the manufacturer’s instructions. MLST was performed by PCR amplification and DNA sequencing of the *cpn*60, *dpr*, *recA*, *aroA*, *thrA*, *gki*, and *mutS* genes as described (*8*). For each isolate, the alleles at each of the 7 loci defined the ST. MLST information in the *S. sui*s database (http://ssuis.mlst.net) identified the phylogenetic position of strains. eBURST software (*14*) was used to identify *S. suis* clonal complexes and to display the overall structure of the population.

### PCRs for Virulence Markers and Pili Cluster Genes

Amplification of *sly*, *mrp*, and *epf* genes was performed by PCR as described (*6*). Genes in the *srtF* and *srtG* pilus clusters were amplified by PCR by using the primers and conditions described by Takamatsu et al. (*13*).

### MRP, EF, and Pili Expression and Hemolysis Assays

*S. suis* strains were grown in Todd-Hewitt broth at 37°C (at 28°C for detection of the *srtG* pilus). Bacteria were harvested by centrifugation during the late exponential phase of growth, and supernatants were concentrated 10-fold by using Ultrafree-MC centrifugal filters (Millipore Corp., Bedford, MA, USA). Expression of extracellular factor (EF) and muramidase-released protein (MRP) was determined by Western blotting of the concentrated supernatant fraction by using monoclonal antibodies against MRP or EF, as described (*15*). Mutanolysin extracts were prepared from pelleted bacteria as described (*16**,**17*) and used to detect pili encoded by the *srtF* and *srtG* pilus clusters by Western blotting with antibodies directed against the major subunit of each pilus (*16**,**17*). The ability of strains to lyse horse erythrocytes (an indication of the production by the strains of the hemolysin known as suilysin, SLY) was determined as described (*6*).

### Experimental Infection of Mice

All animal experiments followed the guidelines of the Canadian Council on Animal Care and were approved by the Ethics Committee, Université de Montréal. We used a validated CD1 mouse infection model (*18*). In a first experiment, 60 female 6-week-old mice (Charles River Laboratories, Wilmington, MA, USA) were divided in 4 groups. Group 1 was inoculated with ST1 strain P1/7; groups 2 and 3 received ST25 strains 89-1591 and 1085543, respectively. Group 4 received ST28 strain 1088563. The inocula (5 × 10^7^ CFU/animal) were delivered intraperitoneally. Mice were monitored 3×/d for 10 days for clinical signs and assigned clinical scores as described (*18*). Blood was collected daily from the tail vein (5 μL) and at necropsy by cardiac puncture and used to evaluate bacterial load by plating onto sheep blood agar plates and enumeration. Colonization of the liver and spleen of infected animals was evaluated at necropsy as described (*18*). A second experiment was performed essentially as described above, but the mice received a 10-fold higher dose of ST28 strains 1088563, 1054471, and 1097205. In this second experiment, groups contained 5 mice.

## Results

Most of the 64 strains from North America were ST28 (n = 33) or ST25 (n = 28). Together, these 2 STs accounted for 95% of all *S. suis* serotype 2 strains from North America that were investigated (Table 1). However, a higher ST28 prevalence was true only for the United States; most strains from Canada were ST25. The remaining 3 strains belonged to ST1, which is commonly found in Europe and Southeast Asia.

**Table 1:** STs identified among the *Streptococcus suis* serotype 2 isolates from North America*

Country	No. strains	ST1	ST25	ST28
Canada	44	0	26	18
United States	20	3	2	15
Total	64	3	28	33

*ST, sequence type.

### Nonrandom Association between STs and Expression of Virulence Markers

SLY (encoded by the *sly* gene), MRP (*mrp* gene), and EF (*epf* gene) are virulence markers that have been used in elaborated genotypic and phenotypic schemes to try to predict the virulence of a given *S. suis* strain (*1**,**19*). For example, Silva et al. designed a multiplex PCR test that can discriminate between at least 6 naturally occurring genetic variants of *mrp*, named *mrp^s^*, *mrp*, mrp*, mrp**, mrp***, and mrp**** (*20*). We investigated possible associations between STs and these widely used markers in our collection of *S. suis* serotype 2 strains from North America. To assess whether associations found are independent of the geographic origin of the strains, we included 32 described (*12**,**13*) *S. suis* serotype 2 strains of STs 28, 25, and 1 isolated in Japan and Thailand (Table A1).

Independently of geographic origin, we found clear, nonrandom associations between STs and expression of virulence markers. All but 2 ST1 strains had the phenotype SLY+MRP+ EF+. All ST25 strains were SLY−MRP−EF− and all ST28 strains were SLY−MRP (or its variants)+ EF− (Table 2). Most ST1 strains had an *sly*+*mrp*+*epf*+ genotype, in agreement with results of previous reports (*11**–**13*). ST25 and ST28 strains had an *sly*− genotype and, with the exception of 3 ST28 strains, an *epf*− genotype. No clear relationships were found between ST25 strains and a particular *mrp* gene variant genotype. All but 3 ST25 strains were positive by PCR for 1 *mrp* gene variant, yet none of these strains expressed the protein (Table 2). In a recent report, all *mrp*+/MRP− strains that were investigated (of various *S. suis* serotypes) had truncations or point mutations in the *mrp* gene that prevented expression of MRP (*6*). Although we have not sequenced the *mrp* gene in our collection of strains, we hypothesize that similar genetic rearrangements are likely to explain the *mrp*+/MRP− results we observed in ST25 strains in this study. Three *mrp* gene variants were associated with ST28, although variant *mrp* was the most prevalent (85%) among this ST.

**Table 2:** Association of *Streptococcus suis* serotype 2 STs and commonly used virulence markers in isolates from North America†

ST	No. strains	Presence of factor-encoding gene									Phenotype		
		*sly*	*mrp* variant‡						*epf*				
			*mrp*	*mrp^s^*	mrp*	mrp**	mrp***	ND			Hemolysis§	MRP¶	EF
1	11	11	9	0	0	0	0	2	11		11	9	11
25	36	0	0	1	1	8	23	3	0		0	0	0
28	49	0	42	6	1	0	0	0	3		0	49	0

†ST, sequence type; SLY, suilysin; MRP, muraminidase-released protein; ND, no amplification of the *mrp* gene was detected by PCR under the conditions used; EF, extracellular factor. ‡Variants of the *mrp* gene are those described by Silva et al. (*20*). §Hemolysis of horse erythrocytes by the strains was considered to be an indication of the expression of SLY. ¶Molecular mass MRP variants identified by Western blotting were in agreement with those expected on the basis of the *mrp* gene variant identified by PCR.

### Nonrandom Association between STs and Expression of Pili

Takamatsu et al. reported associations between particular STs and the presence or absence of putative pilus gene clusters, designated *srtBCD*, *srtE*, *srtF*, and *srtG* clusters (*13*). All ST25 and ST28 strains investigated by these authors were positive by PCR for all genes in the *srtF* and *srtG* pilus clusters (*13*). Consistently, we found that all ST25 and ST28 strains in our collection were positive for all genes in these 2 pilus clusters (Table 3). Furthermore, by using specific antibodies directed against the major pilin subunits (*16**,**17*), we identified a clear, nonrandom association between ST28 strains and expression of both pili (Table 3). However, although all ST25 strains expressed the *srtG* pilus, none produced the *srtF* pilus (Table 3).

**Table 3:** Association of *Streptococcus suis* serotype 2 STs and *srtF* and *srtG* pilus clusters in isolates from North America*

ST	No. strains	*srtF* pilus cluster						*srtG* pilus cluster			
		Gene†				Pili expression‡, Sfp1		Gene†			Pili expression, Sgp1‡
		*srtF*	*sfp1*	*sfp2*	*sipF*			*srtG*	*sgp1*	*sgp2*	
1	11	11	11	11	11	11		3	3	3	0
25	36	36	36	36	36	0		36	36	36	36
28	49	49	49	49	49	46		49	49	49	47

*ST, sequence type. †The presence of the genes was detected by PCR by using primers and conditions described by Takamatsu et al. (*13*). ‡Expression of pili encoded by the *srtF* and *srtG* pilus clusters was performed by Western blotting by using described antibodies directed against the major subunits of these structures (*16**,**17*).

It has been shown that one ST25 isolate from Canada, which does not have a discrete *srtF* pilus cluster and is unable to express the *srtF* pilus, is nonetheless PCR positive for each of the individual *srtF* genes because PCR amplicons can be generated from homologs of these genes found at various genome locations (*13**,**16*). We hypothesized that the ST25 strains analyzed in our study have a genetic organization similar to that ST25 isolate. Consistent with our hypothesis, our attempts to amplify the *srtF* pilus cluster in ST25 strains by using a primer pair annealing to the first and last gene of the *srtF* cluster were unsuccessful (data not shown). All the ST1 strains had the *srtF* cluster genes but, with the exception of 3 strains, not the *srtG* cluster genes. When we assessed the pilus phenotype by Western blotting, all ST1 strains expressed the *srtF* pilus but none expressed the *srtG* pilus (Table 3). The reason(s) the 3 ST1 strains that have the *srtG* cluster genes do not express the corresponding pilus are currently under investigation.

### Mouse Infection Model

**Figure 1:**
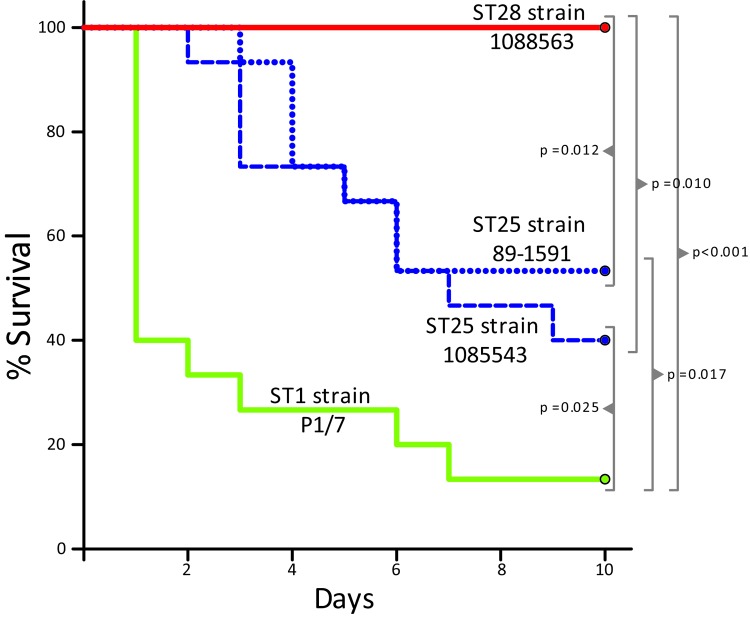
Survival of CD1 mice inoculated with *Streptococcus suis* strains of different sequence types (STs). Most animals that received the ST1 strain P1/7 died from septicemia during the first 3 days of the trial. Several animals in this group died from meningitis from day 6 postinfection. Two groups of mice received ST25 strains 89–1591 and 1085543, respectively. Survival of mice in these 2 groups was higher than in the group that received the ST1 strain. However, >40% of the animals in the 89–1591 group and 60% of the animals in the 1085543 group died or were killed for ethical reasons before the end of the trial. In strong contrast, all 15 mice in the ST28 strain group survived the trial. Significant differences in survival were noted between groups (log-rank test, p values indicated in the figure body).

Inasmuch as the MLST data showed that more than half of the strains from North America analyzed were ST28 and the second most represented ST was ST25, we performed a comparison of the virulence of representative ST25 and ST28 strains by using a standardized mouse infection model (*18*). For comparison, we included the well-characterized and highly virulent ST1 strain P1/7. Most mice in the ST1 group showed severe clinical signs of septicemia, such as depression, swollen eyes, weakness, and prostration during the first 24 hours postinoculation. Several mice died of septicemia during the first 2 days of the trial, and the remaining animals were humanely killed for ethical reasons at day 3 postinoculation (Figure 1). *S. suis* was isolated in pure cultures at high titers (>1 × 10^7^ CFU/mL) from blood samples and organs, such as the liver and spleen, of septicemic animals in the ST1 group (>1 × 10^7^ CFU/0.5 g of tissue in most animals).

**Figure 2:**
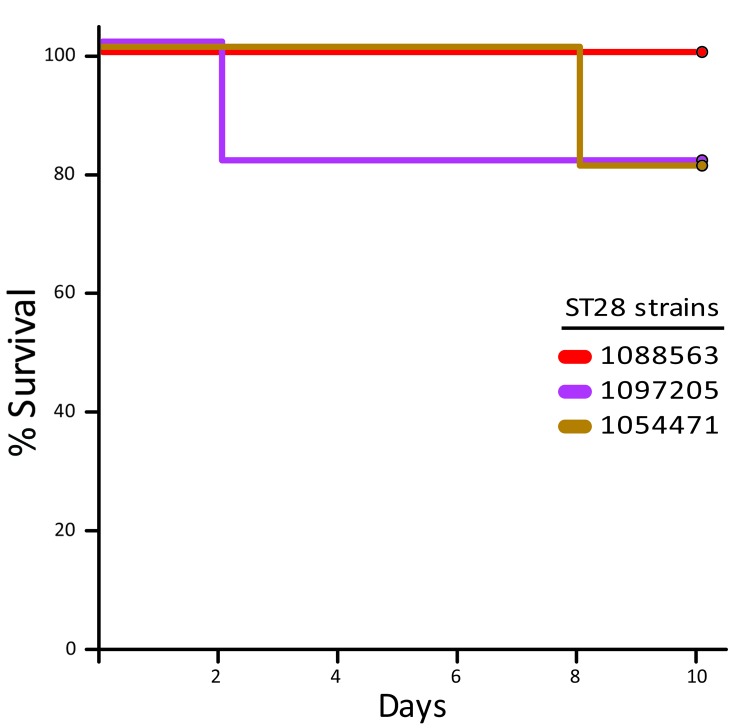
Survival of CD1 mice inoculated with the different *Streptococcus suis* sequence type 28 strains from North America. In this experiment, the infectious dose was 1 × 10^8^ CFU/animal, 10-fold higher than in the previous experimental inoculation. Doses were intraperitoneally injected into the animals. No significant differences were found between groups.

The virulence of ST25 strains was intermediate. They caused moderate clinical signs and relatively low mortality among inoculated mice (Figure 1). Statistical analysis demonstrated that ST25 strains were significantly less virulent than ST1 strains. However, ST25 strains were significantly more virulent than ST28 strains. In fact, no mice in the ST28 group died (Figure 1) or showed clinical signs associated with *S. suis* infection, with the exception of slight depression immediately after inoculation, which subsided after 24 hours postinoculation. Bacteria could not be isolated from the blood of most mice in this group >48 hours postinoculation, and we could not isolate *S. suis* from different organs at necropsy (results not shown). Given this surprising absence of clinical signs, we repeated the experiment by inoculating 3 additional groups of 5 mice each with the previously used and 2 other ST28 strains by using an infective dose that was 10-fold higher than the one previously used. Despite this increased infective dose, similar low virulence was observed for ST28 strains (Figure 2).

## Discussion

In this article, we show that most *S. suis* isolates from North America belong to ST28 and ST25 and that strains of these STs are significantly less virulent than ST1 strains. Although ST28 strains were essentially nonvirulent for mice, ST25 strains were of intermediate virulence and able to induce severe disease.

With a population of ≈115 million pigs, Canada and the United States combined are second only to the People’s Republic of China in terms of swine production. Although *S. suis* infections are a main cause of postweaned piglet deaths in North America, the prevalence of *S. suis* serotype 2 strains is much lower on this continent than in other regions of the world (*6**,**7*). We show here that in North America the most common STs among *S. suis* serotype 2 strains are ST28 and ST25. By using a mouse infection model, we also show that *S. suis* serotype 2 ST28 and ST25 strains are of lower virulence than ST1 strains. In contrast to Europe and Asia, where >60% of virulent serotype 2 isolates are ST1 (*21**–**23*), in North America only a small percentage (5%) of strains belonged to this more virulent ST.

Only 3 cases of *S. suis* serotype 2 in locally infected humans have been reported in North America (*5*). Our results suggest that this low prevalence of human infections might be connected to the lower virulence of the circulating serotype 2 strains among the swine population in North America. In addition to a low prevalence of ST1 strains, we did not find any strains in our collection from North America belonging to STs 101, 102, 103, and 104, which are agents of human disease in Thailand (*12*). On the basis of its low frequency of isolation, we speculate that the ST1 strains we identified were introduced in North America by importation of animals. Human travel might also contribute to dissemination of ST1 strains, as exemplified by a reported case of human *S. suis* meningitis caused by an ST1 strain involving a patient who contracted *S. suis* in the Philippines but in whom clinical signs appeared only after he returned to the United States (*24*). The deadly human outbreaks in Asia caused by ST1 complex strains (*2**–**5*) and the fact that ST1 strains are replacing at a fast pace STs of lower virulence and causing human disease in countries such as Vietnam and Thailand (*21**,**25**,**26*) highlight that maintaining a low prevalence of ST1 strains among the swine population in North America is crucial for animal and human health. Of note, the only locally acquired human infection in the United States described so far (*27*) was caused by an ST1 strain (M. Gottschalk, unpub. data).

Another concern for the swine industry and for public health authorities is the presence in North America of *S. suis* ST25 strains. Many human cases reported in Thailand and 2 cases in Canada of *S. suis* serotype 2 disease were caused by ST25 strains (*5**,**12*). On the other hand, we found that strains of the most prevalent ST28 are of low virulence. Two strains shown here to be ST28 (1330 and 0891; see Table A1) had been reported as nonvirulent *S. suis* serotype 2 (*8*). No human *S. suis* cases attributable to ST28 strains have been reported in North America. However, all ST28 strains included in this study were isolated from diseased pigs, and 1 human case in Japan and 1 human case in Thailand were caused by ST28 strains (*9**,**12*). Nonvirulent *S. suis* strains have been hypothesized to cause disease in immunocompromised animals or humans who have a concurrent infection with another bacterial or viral pathogen(s) (*5*). Porcine *S. suis* infections in North America are usually associated with a concomitant infection with the porcine respiratory and reproductive virus (*1*). We do not know the immunologic status of the animals from which the ST28 strains were isolated to test the aforementioned hypothesis. Toward this goal, however, we are developing a co-infection model of *S. suis* and porcine respiratory and reproductive virus.

Our results provide evidence that genotyping schemes based on *sly*, *mrp*, *epf*, and pilus cluster genes, although useful in discriminating highly virulent ST1 strains from other groups (*8**,**13**,**20*), are of limited use in differentiating between ST25 and ST28 strains. Although not ideal because protein expression levels may be affected by many factors, typing methods based on protein expression of these markers might a priori differentiate these STs of different virulence. The fact that ST25 strains do not express MRP or the *srtF* pili, yet they are more virulent than ST28 strains, further demonstrates the dispensability of these factors for the full virulence of *S. suis* (*16**,**28*). Our results also highlight that subunit vaccines based on purified MRP or *srtF* pilus subunits might be of little use to counter *S. suis* infections caused by ST25 strains.

Our work provides more support to the longstanding hypothesis that *S. suis* serotype 2 strains in North America are of lower virulence than strains from Eurasia. However, we do not yet understand the reasons for this lower virulence. The genome sequences of several *S. suis* serotype 2 ST1 and an ST25 strains have been published or made available (*25**,**29**,**30*). Genome sequencing of a larger number of additional *S. suis* strains of these and other STs could help elucidate the genetic basis of virulence differences among strains of this swine pathogen and zoonotic agent.

## Appendix

**Table A1:** *Streptococcus suis* serotype 2 strains used in study of North American isolates and summary of results†

Strain name	Country	Province/state/ prefecture	Isolation date	ST	Genotype												Phenotype					Host	Tissue/ disease
					*gdh*	*mrp*‡	*sly*	*epf*	*sipF*	*sfp1*	*sfp2*	*srtf*	*sgp1*	*sgp2*	*srtG*		MRP	SLY	EF	Sfp1	Sgp1		
P1/7	United Kingdom	No data	1981	1	+	+	+	+	+	+	+	+	–	–	–		+	+	+	+	–	Pig	Meningitis
1043248	Canada	Quebec	2007 Jan	25	+	***	–	–	+	+	+	+	+	+	+		–	–	–	–	+	Pig	Brain
1043629	Canada	Quebec	2007 Feb	25	+	***	–	–	+	+	+	+	+	+	+		–	–	–	–	+	Pig	Lung
1044423	Canada	Ontario	2007 Jun	25	+	***	–	–	+	+	+	+	+	+	+		–	–	–	–	+	Pig	No data
1053253	Canada	Manitoba	2008 Jan	25	+	***	–	–	+	+	+	+	+	+	+		–	–	–	–	+	Pig	Bronchious
1085543	Canada	Quebec	2008 Jan	25	+	***	–	–	+	+	+	+	+	+	+		–	–	–	–	+	Pig	Meninges
1054470	Canada	Quebec	2007 Jun	25	+	***	–	–	+	+	+	+	+	+	+		–	–	–	–	+	Pig	Lung
1058691	Canada	Manitoba	2007 Aug	25	+	***	–	–	+	+	+	+	+	+	+		–	–	–	–	+	Pig	Meninges
1063930	Canada	Ontario	2007 Mar	25	+	***	–	–	+	+	+	+	+	+	+		–	–	–	–	+	Pig	No data
1064496	Canada	Quebec	2007 Jun	25	+	***	–	–	+	+	+	+	+	+	+		–	–	–	–	+	Pig	Brain
1055923	Canada	Saskatchewan	2007 Aug	25	+	***	–	–	+	+	+	+	+	+	+		–	–	–	–	+	Pig	Bronchious
1072913	Canada	Quebec	2007 Jan	25	+	***	–	–	+	+	+	+	+	+	+		–	–	–	–	+	Pig	Pleura
1074055	Canada	Quebec	2008 Feb	25	+	–	–	–	+	+	+	+	+	+	+		–	–	–	–	+	Pig	Meningitis
1078217	Canada	Ontario	2008 Feb	25	+	***	–	–	+	+	+	+	+	+	+		–	–	–	–	+	Pig	Brain
1078679	Canada	Ontario	2008 Feb	25	+	***	–	–	+	+	+	+	+	+	+		–	–	–	–	+	Pig	Lung
1084568	Canada	Quebec	2008 Mar	25	+	*	–	–	+	+	+	+	+	+	+		–	–	–	–	+	Pig	Brain
1086117	Canada	Manitoba	2008 Apr	25	+	s	–	–	+	+	+	+	+	+	+		–	–	–	–	+	Pig	Endocardium
1087028	Canada	Manitoba	2008 May	25	+	***	–	–	+	+	+	+	+	+	+		–	–	–	–	+	Pig	Brain
1088904	Canada	Quebec	2008 May	25	+	***	–	–	+	+	+	+	+	+	+		–	–	–	–	+	Pig	Meningitis
1091168	Canada	Quebec	2008 Jun	25	+	***	–	–	+	+	+	+	+	+	+		–	–	–	–	+	Pig	Meningitis
1093400	Canada	Ontario	2008 Jun	25	+	***	–	–	+	+	+	+	+	+	+		–	–	–	–	+	Pig	Brain
1097204	Canada	Quebec	2008 Jun	25	+	***	–	–	+	+	+	+	+	+	+		–	–	–	–	+	Pig	Lung
1098986	Canada	Quebec	2008 Jul	25	+	***	–	–	+	+	+	+	+	+	+		–	–	–	–	+	Pig	Lung
1102864	Canada	Quebec	2008 Aug	25	+	***	–	–	+	+	+	+	+	+	+		–	–	–	–	+	Pig	Multiple
1102337	Canada	Quebec	2008 Jul	25	+	***	–	–	+	+	+	+	+	+	+		–	–	–	–	+	Pig	Meningitis
1111483	Canada	Quebec	2008 Sep	25	+	***	–	–	+	+	+	+	+	+	+		–	–	–	–	+	Pig	Brain
1084708	Canada	Ontario	2008 Apr	28	+	+	–	–	+	+	+	+	+	+	+		+	–	–	+	+	Pig	Pleura
1085273	Canada	Quebec	2008 Apr	28	+	s	–	–	+	+	+	+	+	+	+		+	–	–	+	+	Pig	Nasal sample
1054471	Canada	Manitoba	2007 Jul	28	+	+	–	–	+	+	+	+	+	+	+		+	–	–	+	+	Pig	Brain
1064089	Canada	Saskatchewan	2007 Sep	28	+	s	–	–	+	+	+	+	+	+	+		+	–	–	+	+	Pig	Tonsil
1057906	Canada	Saskatchewan	2007 Jul	28	+	s	–	–	+	+	+	+	+	+	+		+	–	–	+	+	Pig	Brain
1064773	Canada	Quebec	2007 Oct	28	+	+	–	–	+	+	+	+	+	+	+		+	–	–	–	+	Pig	Lung
1077008	Canada	Quebec	2008 May	28	+	*	–	–	+	–	+	+	+	+	+		+	–	–	–	+	Pig	Endocardium
1082563	Canada	Quebec	2008 Apr	28	+	+	–	–	+	+	+	+	+	+	+		+	–	–	+	+	Pig	Endocardium
1088563	Canada	Quebec	2008 May	28	+	+	–	–	+	+	+	+	+	+	+		+	–	–	+	+	Pig	Brain
1089976	Canada	Quebec	2008 May	28	+	s	–	–	+	+	+	+	+	+	+		+	–	–	+	+	Pig	Endocardium
1090152	Canada	Ontario	2008 Jun	28	+	+	–	–	+	+	+	+	+	+	+		+	–	–	+	+	Pig	Lung
1090686	Canada	Saskatchewan	2008 Jun	28	+	+	–	–	+	+	+	+	+	+	+		+	–	–	+	+	Pig	No data
1097205	Canada	Ontario	2008 Jun	28	+	+	–	–	+	+	+	+	+	+	+		+	–	–	+	+	Pig	Brain
1097811	Canada	Ontario	2008 Jun	28	+	s	–	–	+	+	+	+	+	+	+		+	–	–	+	+	Pig	Liver/lung/kidney
1110359	Canada	Quebec	2008 Sep	28	+	s	–	–	+	+	+	+	+	+	+		+	–	–	+	+	Pig	Pleura
1111490	Canada	Manitoba	2008 Sep	28	+	+	–	–	+	+	+	+	+	+	+		+	–	–	+	+	Pig	Lung
89-1591	Canada	Manitoba	1989	25	+	***	–	–	+	+	+	+	+	+	+		–	–	–	–	+	Pig	Septicemia, meningitis
1330	Canada	Quebec	1995	28	+	+	–	–	+	+	+	+	+	+	+		+	–	–	+	+	Pig	Lung
0891	Canada	Quebec	1995	28	+	+	–	–	+	+	+	+	+	+	+		+	–	–	+	+	Pig	Lung
NIAH11433	Japan	No data	1989	1	+	+	+	+	+	+	+	+	–	–	–		+	+	+	+	–	Pig	Meningitis
DAT261	Japan	Gunma	1993 Jan	1	+	+	+	+	+	+	+	+	–	–	–		+	+	+	+	–	Pig	Multiple serositis, pneumonia
DAT264	Japan	Gunma	1994 Jun	1	+	+	+	+	+	+	+	+	–	–	–		+	+	+	+	–	Pig	Meningitis
DAT229	Japan	Aichi	2006 Jan	1	+	+	+	+	+	+	+	+	–	–	–		+	+	+	+	–	Pig	Endocarditis
DAT273	Japan	Nagasaki	2002 Feb	1	+	–	+	+	+	+	+	+	–	–	–		–	+	+	+	–	Human	Meningitis
DAT292	Japan	Okinawa	2005 Dec	28	+	+	–	–	+	+	+	+	+	+	+		+	–	–	+	+	Pig	Healthy carrier
DAT242	Japan	Ibaraki	1990 Jan	28	+	+	–	–	+	+	+	+	+	+	+		+	–	–	+	+	Pig	Meningitis
DAT245	Japan	Ibaraki	1995 Jan	28	+	+	–	–	+	+	+	+	+	+	+			–	–	+	+	Pig	Meningitis
DAT246	Japan	Ibaraki	1996 Apr	28	+	+	–	–	+	+	+	+	+	+	+		+	–	–	+	+	Pig	Septicemia
DAT251	Japan	Ishikawa	1991 Jun	28	+	+	–	–	+	+	+	+	+	+	+		+	–	–	+	+	Pig	Liver
DAT253	Japan	Ishikawa	1993 Aug	28	+	+	–	–	+	+	+	+	+	+	+		+	–	–	+	+	Pig	Brain
DAT254	Japan	Ishikawa	1996 Jul	28	+	+	–	–	+	+	+	+	+	+	+		+	–	–	+	+	Pig	Brain
DAT255	Japan	Niigata	No data	28	+	+	–	–	+	+	+	+	+	+	+		+	–	–	+	+	Pig	Septicemia
DAT256	Japan	Niigata	No data	28	+	+	–	–	+	+	+	+	+	+	+		+	–	–	+	+	Pig	Meningitis
DAT259	Japan	Gunma	1992 Nov	28	+	+	–	–	+	+	+	+	+	+	+		+	–	–	+	+	Pig	Multiple serositis, pneumonia
DAT260	Japan	Gunma	1992 Dec	28	+	+	–	–	+	+	+	+	+	+	+		+	–	–	+	+	Pig	Meningitis
DAT272	Japan	Yamagata	No data	28	+	+	–	–	+	+	+	+	+	+	+		+	–	–	+	+	Pig	Brain
DAT274	Japan	Kumamoto	1994 Mar	28	+	+	–	–	+	+	+	+	+	+	+		+	–	–	+	+	Pig	Endocarditis
DAT281	Japan	Kumamoto	No data	28	+	+	–	–	+	+	+	+	+	+	+		+	–	–	+	–	Pig	Meningitis
DAT285	Japan	Ibaraki	No data	28	+	+	–	–	+	+	+	+	+	+	+		+	–	–	+	+	Pig	Endocarditis
MNCM01	Thailand	Chiang Mai	2000 Jun	1	+	+	+	+	+	+	+	+	–	–	–		+	+	+	+	–	Human	Endocarditis
MNCM06	Thailand	Chiang Mai	2000 Aug	1	+	+	+	+	+	+	+	+	–	–	–		+	+	+	+	–	Human	Meningitis
MNCM16	Thailand	Chiang Mai	2000 Nov	1	+	+	+	+	+	+	+	+	–	–	–		+	+	+	+	–	Human	Meningitis
MNCM04	Thailand	Chiang Mai	2000 Aug	25	+	**	–	–	+	+	+	+	+	+	+		–	–	–	–	+	Human	Meningitis
MNCM10	Thailand	Chiang Mai	2000 Sep	25	+	**	–	–	+	+	+	+	+	+	+		–	–	–	–	+	Human	Septicemia
MNCM24	Thailand	Chiang Mai	2001 Aug	25	+	**	–	–	+	+	+	+	+	+	+		–	–	–	–	+	Human	Endocarditis
MNCM26	Thailand	Chiang Mai	2001 Nov	25	+	**	–	–	+	+	+	+	+	+	+		–	–	–	–	+	Human	Meningitis, endocarditis
MNCM51	Thailand	Chiang Mai	2002 Oct	25	+	**	–	–	+	+	+	+	+	+	+		–	–	–	–	+	Human	Septicemia
MNCM55	Thailand	Chiang Mai	2002 Dec	25	+	**	–	–	+	+	+	+	+	+	+		–	–	–	–	+	Human	Septic shock
LPH4	Thailand	Lamphun	2001 May	25	+	**	–	–	+	+	+	+	+	+	+		–	–	–	–	+	Human	Septicemia
LPH12	Thailand	Lamphun	2002 Mar	25	+	**	–	–	+	+	+	+	+	+	+		–	–	–	–	+	Human	Septic shock
MNCM43	Thailand	Chiang Mai	2002 Jun	28	+	+	–	–	+	+	+	+	+	+	+		+	–	–	+	+	Human	Endocarditis
MGGUS1	United States	Minnesota	2003 Mar	1	+	–	+	+	+	+	+	+	+	+	+		–	+	+	+	–	Pig	No data
MGGUS2	United States	Wisconsin	2003 Feb	1	+	+	+	+	+	+	+	+	+	+	+		+	+	+	+	–	Pig	Brain
MGGUS3	United States	Iowa	2003 May	1	+	+	+	+	+	+	+	+	+	+	+		+	+	+	+	–	Pig	Brain
MGGUS4	United States	Iowa	2005 May	25	+	–	–	–	+	+	+	+	+	+	+		–	–	–	–	+	Pig	Septicemia
MGGUS5	United States	Nebraska	No data	25	+	–	–	–	+	+	+	+	+	+	+		–	–	–	–	+	Pig	Septicemia
MGGUS6	United States	Minnesota	No data	28	+	+	–	+	+	+	+	+	+	+	+		+	–	–	+	+	Pig	No data
MGGUS7	United States	Kansas	1995	28	+	+	–	+	+	+	+	+	+	+	+		+	–	–	+	+	Pig	No data
MGGUS8	United States	Kansas	1995	28	+	+	–	+	+	+	+	+	+	+	+		+	–	–	+	+	Pig	No data
MGGUS9	United States	Oklahoma	2003 Dec	28	+	+	–	–	+	+	+	+	+	+	+		+	–	–	–	+	Pig	Heart
MGGUS10	United States	Illinois	2005 May	28	+	+	–	–	+	+	+	+	+	+	+		+	–	–	+	+	Pig	Lung
MGGUS11	United States	Virginia	2005 May	28	+	+	–	–	+	+	+	+	+	+	+		+	–	–	+	+	Pig	Lung
MGGUS12	United States	Iowa	2005 May	28	+	+	–	–	+	+	+	+	+	+	+		+	–	–	+	+	Pig	Lung
MGGUS13	United States	Oklahoma	2004 Oct	28	+	+	–	–	+	+	+	+	+	+	+		+	–	–	+	+	Pig	Liver/lung/ kidney
MGGUS14	United States	Oklahoma	No data	28	+	+	–	–	+	+	+	+	+	+	+		+	–	–	+	+	Pig	Lung
MGGUS15	United States	Oklahoma	No data	28	+	+	–	–	+	+	+	+	+	+	+		+	–	–	+	+	Pig	Liver/lung/ kidney
MGGUS16	United States	Nebraska	2004 Jun	28	+	+	–	–	+	+	+	+	+	+	+		+	–	–	+	+	Pig	Lung
MGGUS17	United States	Oklahoma	2004 Dec	28	+	+	–	–	+	+	+	+	+	+	+		+	–	–	+	+	Pig	Spleen
MGGUS18	United States	North Carolina	No data	28	+	+	–	–	+	+	+	+	+	+	+		+	–	–	+	+	Pig	Liver/lung/ kidney
MGGUS19	United States	Nebraska	No data	28	+	+	–	–	+	+	+	+	+	+	+		+	–	–	+	+	Pig	Liver/lung/ kidney
MGGUS20	United States	Kentucky	2004 Jan	28	+	+	–	–	+	+	+	+	+	+	+		+	–	–	+	+	Pig	Spleen

†ST, sequence type; MRP, muramidase-released protein; SLY, suilysin; EF, extracellular protein factor; +, positive; −, negative. ‡The identified *mrp* variant (*mrp^s^*, *mrp*, *mrp*,* *mrp**,* *mrp****, *mrp*****) is indicated.
